# Supplementary material for: Content-rich biological network constructed by mining PubMed abstracts
Source: BMC Bioinformatics. 2004 Oct 8;5:147. doi: 10.1186/1471-2105-5-147 (PMC528731; doi:10.1186/1471-2105-5-147)
Supplement: Additional File 2 — The original results of the above study (non-essential files are deleted to keep the file size under the limit set by BMC bioinformatics). [file 1471-2105-5-147-S2.bz2 › chilibotAdditionalFile2/dip10/295ID3304349E1137/html/SUCD_SUCC.html]

 


 **SUCD** and **SUCC** 
  
Found 6 abstracts in PubMed, retrieved 6.  
 

 What does Google say? 
 PDF only 
| .edu only 

---

**Interactive relationship** (e.g. stimulation, inhibition, etc)

**Neutral relationship**- The stop codon of the  **sucC**  gene overlaps the  **sucD**  initiation codon by a single nucleotide, indicating close translational coupling of the  **sucC**  and  **sucD**  genes.  Ref: 3002435 Biochemistry, 1985

**Non-interactive relationship** (e.g. studied together, co-existance, homology, etc.)

- The  **sucC**  and  **sucD**  genes are separated from the sucA and sucB genes by a 273 base pair segment containing four palindromic units, but they appear to be expressed from a sucABCD read through transcript that extends from the suc promoter to a potential rho independent terminator at the distal end of  **sucD** .  Ref: 3002435 Biochemistry, 1985
- The newly defined genes express polypeptides of 41 kDa  **sucC**  and 31 kDa  **sucD** , corresponding to the beta and alpha subunits of succinyl CoA synthetase, respectively.  Ref: 3543212 J Gen Microbiol, 1986
- The primary structure of the succinyl CoA synthetase of Escherichia coli has been deduced from the nucleotide sequence of a 2451 base pair segment of DNA containing the corresponding  **sucC**  beta subunit and  **sucD**  alpha subunit genes.  Ref: 3002435 Biochemistry, 1985
- The succinyl CoA synthetase of Escherichia coli is encoded by two genes,  **sucC**  beta subunit and  **sucD**  alpha subunit, which are distal genes in the sucABCD operon.  Ref: 2548486 Biochem J, 1989
- The combination of highly efficient transcriptional and translational signals on these vectors allowed high level expression of sequences encoding human interferon beta and interleukin 2 and of the E. coli atpA,  **sucC**  and  **sucD**  genes.  Ref: 3038690 Gene, 1987
- The succinyl CoA synthetase activities of strains harbouring plasmids containing the  **sucC**  and  **sucD**  genes were amplified some fourfold.  Ref: 3543212 J Gen Microbiol, 1986
- Mutation of either  **sucC**  or  **sucD**  prevents expression of 2 oxoglutarate dehydrogenase sucAB.  Ref: 9245810 Microbiology, 1997
- We used a PCR approach to clone fragments of the putative yhhF,  **sucC** ,  **sucD** , cypH, pbpB, murE, pbpC, soxR, ftsA, ftsZ and envA genes.  Ref: 8581173 Microbiology, 1996
